# Olfactory response as a marker for Alzheimer’s disease: Evidence from perceptual and frontal lobe oscillation coherence deficit

**DOI:** 10.1371/journal.pone.0243535

**Published:** 2020-12-15

**Authors:** Mohammad Javad Sedghizadeh, Hadi Hojjati, Kiana Ezzatdoost, Hamid Aghajan, Zahra Vahabi, Heliya Tarighatnia

**Affiliations:** 1 Department of Electrical Engineering, Sharif University of Technology, Tehran, Iran; 2 Department of Geriatric Medicine, Ziaeian Hospital, Tehran University of Medical Sciences, Tehran, Iran; 3 Memory and Behavioral Neurology Division, Roozbeh Hospital, Tehran University of Medical Sciences, Tehran, Iran; Universite de Lyon, FRANCE

## Abstract

High-frequency oscillations of the frontal cortex are involved in functions of the brain that fuse processed data from different sensory modules or bind them with elements stored in the memory. These oscillations also provide inhibitory connections to neural circuits that perform lower-level processes. Deficit in the performance of these oscillations has been examined as a marker for Alzheimer’s disease (AD). Additionally, the neurodegenerative processes associated with AD, such as the deposition of amyloid-beta plaques, do not occur in a spatially homogeneous fashion and progress more prominently in the medial temporal lobe in the early stages of the disease. This region of the brain contains neural circuitry involved in olfactory perception. Several studies have suggested that olfactory deficit can be used as a marker for early diagnosis of AD. A quantitative assessment of the performance of the olfactory system can hence serve as a potential biomarker for Alzheimer’s disease, offering a relatively convenient and inexpensive diagnosis method. This study examines the decline in the perception of olfactory stimuli and the deficit in the performance of high-frequency frontal oscillations in response to olfactory stimulation as markers for AD. Two measurement modalities are employed for assessing the olfactory performance: 1) An interactive smell identification test is used to sample the response to a sizable variety of odorants, and 2) Electroencephalography data are collected in an olfactory perception task with a pair of selected odorants in order to assess the connectivity of frontal cortex regions. Statistical analysis methods are used to assess the significance of selected features extracted from the recorded modalities as Alzheimer’s biomarkers. Olfactory decline regressed to age in both healthy and mild AD groups are evaluated, and single- and multi-modal classifiers are also developed. The novel aspects of this study include: 1) Combining EEG response to olfactory stimulation with behavioral assessment of olfactory perception as a marker of AD, 2) Identification of odorants most significantly affected in mild AD patients, 3) Identification of odorants which are still adequately perceived by mild AD patients, 4) Analysis of the decline in the spatial coherence of different oscillatory bands in response to olfactory stimulation, and 5) Being the first study to quantitatively assess the performance of olfactory decline due to aging and AD in the Iranian population.

## Introduction

Alzheimer’s disease (AD) is the most prevalent type of dementia affecting approximately one individual in 10 in the population older than 65 [1]. Early diagnosis of AD is necessary to ensure that the required clinical and social care are provided for affected individuals [2]. AD is known to be associated with the aggregated deposition of surplus amyloid-beta (Aβ) protein, a product of synaptic activity [3], as plaques causing neurotoxic events such as inflammation and synaptic loss, and other neural degenerations linked to another protein, phosphorylated tau [4].

Accumulated levels of these proteins in the brain have been measured as biomarkers for AD through PET imaging of the brain or sampling the cerebrospinal fluid (CSF) [5, 6]. Several neuropsychological tests have also been introduced to evaluate the mental state of subjects in a clinical exam. Mini-Mental State Exam (MMSE) and Mini-Cog are two examples of such tests, which are used by clinicians to evaluate the cognitive skills of the patients and decide upon further evaluation tests [7–9]. Although no single protocol has been established for large-scale screening of AD, there are proposed frameworks for diagnosis based on a set of biomarkers such as those just mentioned [10]. An update to the National Institute on Aging—Alzheimer’s Association (NIA-AA) Research Framework provides additional flexibility for introducing new biomarkers to allow the results of new measurement modality evaluations in observational studies to establish their value in the clinical assessment of AD [11].

In addition to the mentioned assessment methods, previous studies have shown that olfactory deficit is an early symptom of Alzheimer’s disease [12, 13]. Further studies have demonstrated that standard methods of assessing the olfactory system such as sniffing kits can be helpful in distinguishing mild AD patients from healthy individuals [14–16].

The neurodegenerative processes associated with the deposition of neurofibrillary tangles and amyloid plaques in the brain do not progress in a homogeneous fashion [17], and are more prominent in the medial temporal lobe in the early stage of the disease [18, 19]. Interestingly, the medial temporal lobe is the region where olfactory perception also occurs. Therefore, perception of smells is affected more severely in mild AD patients compared to its decline caused by normal aging, and several studies have suggested that olfactory deficit can be used as a biomarker for early diagnosis of AD [20, 21].

Unlike PET imaging or CSF sampling, measuring the odor perception abilities of patients is an inexpensive and non-invasive procedure. However, the perception of odors highly correlates with the culture of the individuals, and hence, the familiarity of subjects with the employed odorants needs to be considered in defining a smell scoring procedure. The University of Pennsylvania smell identification test (UPSIT) [22] proposes a standard sniffing kit for assessing the olfactory function and has been used for studying the olfactory deficit in mild AD patients [23]. However, some of the odors used in this test may be unfamiliar for non-American societies. To address this issue, researchers have proposed alternative scents in modified sniffing kits to create tests that are suitable for their populations of interest [24–26].

A practical issue in administering sniffing kit tests is that they require the participant’s cooperation. Patients with dementia-like symptoms may have difficulty following the written test questions or the clinician’s instructions or may respond erroneously due to not recalling the name of an odorant they indeed perceived. Also, the way an examiner interacts with the participant may introduce bias towards specific options in the response sheet [36]. To circumvent the interference of non-olfactory related issues in the performance of the tests, methods relying on EEG recording during the presentation of odorants to participants have been proposed. One such technique is the olfactory event-related potential (OERP) test, in which a sequence of odorants is presented to the participant at regular time instances, allowing the EEG response data to be averaged over several trial intervals for reducing noise and enhancing the fidelity of the recorded data. Several studies have focused on the role of OERP test results as an early biomarker for AD. In [27] OERP waveforms were analyzed to extract features for differentiating mild AD patients and an age-matched control group. In a more recent study [28], OERP test was employed to distinguish between AD and mild cognitive impairment (MCI) patients.

Another method for the differential analysis of EEG data of mild AD patients and healthy participants is coherence analysis. Coherence refers to the functional connectivity of different brain regions and is measured by the synchrony of oscillations recorded at different EEG electrodes. Earlier studies have indicated that the coherence of EEG channels can help in the diagnosis of AD [29–31]. These studies showed that the reduction in the functional connectivity of the brain regions is captured as a decrease in the coherence between EEG channels [32]. Some reports have also assessed the relative value of the coherence of EEG channels for different frequency bands in the classification of mild AD patients and healthy participants [33, 34].

In this paper, we examine the characteristics of olfactory response as markers for the diagnosis of AD through the use of both EEG and behavioral olfactory response data. Our EEG analysis comprises an assessment of the statistical significance of the coherence in the EEG data across the spatial domain for different frequency bands. In the behavioral olfactory response data, our approach identifies the best subset of odorants among those in a localized version of the UPSIT kit (Iran-SIT [16]), which significantly contributes to classifying mild AD patients and healthy participants. MMSE scores are also used as reference for evaluating our olfactory-based results. Single-modality regressors are developed employing the significant components identified in each set of olfactory response data (EEG coherence and behavioral UPSIT) separately. The regressors are age-adjusted to account for the decline in the performance caused by normal aging. Furthermore, by employing the statistically significant components from both modalities, we propose a multi-modal classifier of mild AD patients versus healthy participants, which also regresses the olfactory decline due to aging.

## Materials and methods

**Fig 1:**
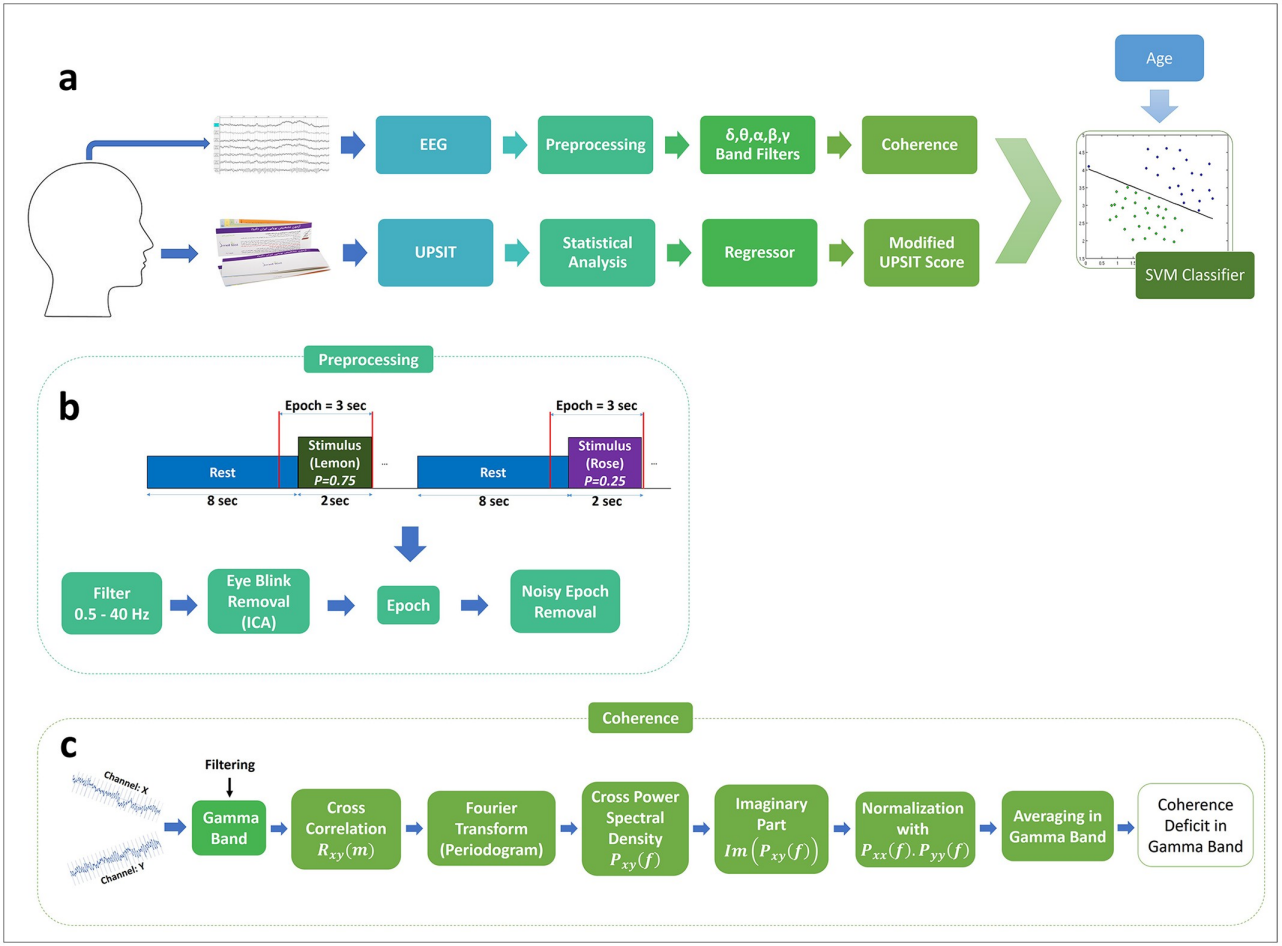
a) An overview of our methodology. The coherence between EEG electrode pairs in different frequency bands and the modified UPSIT (Iran-SIT) score are used as features for training an SVM classifier. The selection of the odorant subset in the UPSIT kit and the significant frequency bands and connections in the EEG records is carried out by statistical analysis. b) EEG response to the sequence of stimuli is pre-processed and epochs are extracted for further analysis. c) A measure of coherence deficit is calculated between each pair of channels for the gamma oscillation band. Similar operations are performed for each of the other oscillatory bands (delta, theta, alpha, beta).

Fig 1 illustrates an overview of the multimodal data analysis methodology used in our study. Details of our experiment’s protocol are available at “protocols.io” under the DOI number: 10.17504/protocols.io.bi2qkgdw.

### Participants

This study was approved by the Review Board of Tehran University of Medical Sciences (Approval ID: IR.TUMS.MEDICINE.REC.1398.524) and all participants gave their written consent to participate in the experiment. Private information of participants including name and date of birth were kept confidential and not used in any of the analyses. Participants were selected among the individuals referring to the memory clinic of Ziaeian Hospital in Tehran with memory performance complaints. All the tests were carried out in the Department of Geriatric Medicine of Ziaeian Hospital. Two expert neuropsychologists assessed all the participants and recorded their smoking history, preferred hand, age, level of education, as well as any past olfactory problem. Demographic and medical history data were also collected for each participant. Statistical analyses suggested that sufficient sample size for comparing AD and healthy groups with a statistical power of 95% and type I error of 5% is about 7 participants for each group. A total of 52 participants were recruited for the study, and after applying exclusion criteria (as described in the next subsection), twenty-four individuals (age = 72.1 ± 9.0, female = 54.25%), including 11 participants with AD (age = 76.6 ± 9.2, female = 64%) and 13 healthy participants (age = 68.2 ± 6.2, female = 46%) were selected for data analysis. The Mini-Mental State Examination (MMSE), the Clock Drawing Test (CDT), and a verbal fluency test were performed. After the neuropsychological assessment, a neurologist examined the participants and conducted the Functional Assessment Scales Test (FAST) [35].

Then, the participants performed the UPSIT examination and after a few minutes of rest, performed the EEG-based olfactory measurement test. Table 1 shows the overall statistics of the participants. Details of the clinical assessment procedure and the MMSE, UPSIT, and EEG-based experiments are described in the following subsections.

**Table 1:** Participant characteristics: P-values denote the separation between healthy participants and mild AD patients in each characteristic.

Characteristic	Healthy (n = 13)	AD (n = 11)	p-value
**Age (years)**	68.2 ± 6.2	76.6 ± 9.2	< 0.05
**Gender, % female**	%46	%64	> 0.4
**Education (years)**	3.36 ± 2.94	4.15 ± 4.70	> 0.4
**MMSE score**	25.8 ± 3.3	15.7 ± 2.9	< 0.0001
**UPSIT score**	15.5 ± 2.8	8.2 ± 3.9	< 0.0001

### Clinical diagnostic assessment

An expert neurologist diagnosed probable Alzheimer’s disease according to the latest guideline of the NIA-AA [36]. AD participants must meet the criteria prescribed for diagnosing dementia as described in [36]. Results of the Mini-Mental State Examination (MMSE) and inquiry about the onset and progressions of the symptoms from the patients and their companions were used by the neurologist to diagnose cognitive impairment. In addition to criteria for dementia, AD participants must also meet the criteria for probable AD dementia. Structural MRI images (1.5 Tesla MR Scanner and a 16-channel HR head coil) were analyzed, and the Medial Temporal Atrophy Scale, White Matter Lesions, and Global Atrophy Scale were used for describing the image. Exclusion criteria were a history of stroke, schizophrenia, major depressive disorders and electroconvulsive therapy (ECT) over the past six months, traumatic brain injury, non-AD neurodegenerative diseases (Parkinson’s disease, Progressive Supranuclear Palsy, Multi-System Atrophy, Cortico-Basal Degeneration), and any history of olfactory pathway disorders. MCI patients were also excluded from this study.

### Mini-Mental State Examination (MMSE)

MMSE [37] is a clinical test commonly used for measuring cognitive impairment. During the MMSE test, different memory skills are evaluated, and a score out of 30 is produced. Based on this score and the education level of the patients, clinicians assess the participant’s cognitive skills.

MMSE consists of the following cognitive test categories: Orientation to time, Orientation to place, Registration, Attention and calculation, Delayed recall, Naming, Repetition, Reading, Writing, Visio-spatial, and Commands. A score is given in each category, and the sum of a participant’s scores in all categories is used as the MMSE score. It should be mentioned that due to the low literacy levels of many of the participants in this study, lower MMSE scores were registered in both AD and healthy control groups.

### University of Pennsylvania Smell Identification Test (UPSIT)

UPSIT and its modified versions have been employed in previous studies for early diagnosis of AD. Due to the culture-specific nature of smell perception, the reliability of these tests has to be evaluated in different populations [2, 38]. Localized versions of the UPSIT test kit have been introduced in countries such as Brazil [24], Turkey [25], Lithuania [26], and Iran [16] (for an exhaustive review refer to [14]).

To this date, no research results based on the UPSIT kit or other olfactory-based tests have been reported for the detection of AD in the Iranian population. The current study is the first to utilize a localized version of the UPSIT test (called Iran-SIT) to diagnose AD in its early stages in Iran.

The test kit consists of 24 odors which are each exposed by scratching its corresponding strip. The list of the odors is included in S1 Table in S1 Appendix. After presenting each scent to the participant, four options to select from are provided, and the participant is asked to identify the closest match among these options to the odor that they perceived. As some participants in the study were not able to read the list of options printed in the kit, either because of vision problems or due to illiteracy, the list of options for each odor presentation was read loudly and clearly to the participants, once before and once after the presentation of each odor.

#### Classification

To assess the results of the UPSIT test, we employed a support vector machine (SVM) classifier with a linear kernel to separate mild AD patients from healthy participants based on the UPSIT score and the age of the participants. Normal aging is known to be a major cause for olfactory deficit and hence, when dealing with the UPSIT or other olfactory test results, it is essential to take into account the effect of aging.

Due to the small size of our dataset, we used 5-fold cross-validation to evaluate the accuracy of the classifier. In this evaluation scheme, the dataset is divided into five equal folds, and each time, the label of one fold is predicted using the model trained on data of the other folds.

#### Statistical analysis of UPSIT

The UPSIT score denotes the number of correct answers for each participant. However, among the twenty-four odors of the test, some are probably more effective at separating Alzheimer’s patients from healthy participants. To determine these significant odors, each participant’s answers were converted to a vector of binary elements in which zeros represent wrong answers and ones indicate correct answers. These vectors were divided into two groups of mild AD patients and healthy participants and then t-test was applied to samples from these groups. By doing this, twenty-four p-values corresponding to the presented odors were obtained. To control the false discovery rate (FDR), the Benjamini-Hochberg method was used [39]. The remaining small p-values (p-value < 0.05) indicate odors that are significant in separating the two participant groups. The Scipy and Statsmodels packages were used for statistical analysis and p-values < 0.05 were considered as significant. The full flow of the statistical analysis for identifying significant odors is illustrated in S1 Fig in S1 Appendix and the values behind the corrected p-values are shown in S2 Table in S1 Appendix.

After identifying the significant odors, we performed a linear regression analysis to obtain a modified UPSIT score as a weighted sum of the significant odor scores. The coefficient of each score in the linear regressor is set to maximize the separability of the mild AD patients and healthy participants.

We fitted a linear regression model to each of the mild AD patients and healthy participant groups using the modified UPSIT score and age. Each regression line demonstrates how the olfactory perception is affected by age in the AD or healthy groups.

### EEG-based olfactory measurement

EEG signals were recorded using a 32-channel Mitsar amplifier. Data were recorded from the Fz, Cz, Pz, and Fp1 electrodes. The Fz, Cz, and Pz channels were chosen based on the results of a similar study [28]. The Fp1 electrode data was used to identify eye movements and eye blinks. The choice of using a limited number of channels in this study was to accommodate for the age and mental condition of many of the participants, so the time to install and confirm the functionality of the electrodes would be kept to a minimum. The channel impedance was maintained under 15 kΩ for each electrode. The EEG sampling rate was 2000 Hz, and electrodes were referenced to the A1 earlobe [40]. EEGLAB was used for data preprocessing [41].

Participants performed an olfactory perception task [42]. During this task, the participant is presented with a sequence of stimuli composed of two different odors, one of which occurring more frequently (standard) and the other being presented rarely (deviant) [43]. Lemon was chosen as the frequent stimulus and Rose as the rare one [28]. Our experimental protocol consisted of a two-second stimulus presentation followed by 8 seconds of rest (pure water) interval. The odors were delivered to the participant using a laboratory olfactometer [44]. The probability of rare stimuli was 0.25 [28]. Each trial (epoch) took 10 seconds, and the whole experiment for each participant consisted of 120 trials and took about 20 minutes. The 90 frequent and 30 rare odors were presented in a random but preset order.

The choice of odors in olfactory experiments is an important issue. When the objective is to test the performance of the olfactory system, odors must be selected so as not to arouse the trigeminal system. This is because the olfactory and the trigeminal systems are interconnected and may interact by intensifying and suppressing each other during exposure to certain stimuli [45]. Therefore, in our experiments, we replaced the eucalyptus odor which was used in other studies [28] with lemon, since eucalyptus excites both the olfactory and trigeminal systems. We also increased the duration of odor presentation to two seconds to allow for regular breathing cycles by the participants (to accommodate for their age and mental condition). The extracted event epochs included one second of pre-stimulus and two seconds of post-stimulus data, following the empirical estimate of the olfactory response latency of about 600–700 milliseconds [46, 47].

#### EEG preprocessing

Steps involved in preprocessing the data and extracting epochs for further processing are shown in Fig 1b. Signals were filtered to 0.5 to 40.5 Hz and downsampled to 200 Hz. Independent Component Analysis (FastICA [48]) was used for eye blink removal using all recorded EEG channels. One component corresponding to eye blinks was removed from the four components, and the rest were projected back to the electrode space. The resulting signals were segmented into epochs as follows. Each epoch contains one second of pre- and two seconds of post-stimulus onset (600 samples with a sampling frequency of 200 Hz). From the entire task which included 120 epochs, the ones corresponding to the frequent (lemon) odor were selected for further processing. For each subject, this set includes about 90 epochs. Finally, heavily artifact-contaminated epochs were excluded using a semi-automated method of rejecting epochs with high peak-to-average ratios and manually inspecting the remaining epochs.

#### Coherence analysis

Fig 1c illustrates the flow of data processing to measure the deficit in the spatial coherence of EEG response for the gamma oscillation band. Since coherence analysis involves nonlinear operations, we first filtered the EEG data in the target band in order to mitigate the effects of other frequency bands. Therefore, for the gamma oscillation band, the preprocessed EEG data were filtered using a bandpass (30.0–40.5 Hz) filter. The upper frequency limit of 40.5 Hz was imposed by the EEG recording system which was programmed to remove higher frequency components including the 50 Hz powerline interference. The coherence analysis method was then applied to the gamma band data for the epochs corresponding to the frequent (lemon) stimuli.

For each pair of connections between the four channels, the imaginary part of coherence (ImCoh) was obtained for each frequency using the following equation:

$$ImCoh(C_{xy}(f))\;=\;\frac{Im{\left(P_{xy}(f)\right)}^{2}}{P_{xx}(f)P_{yy}(f)}$$ (1)

in which *P*_*xy*_(*f*) indicates the cross power spectral density between channels *x* and *y* for frequency *f*, and *P*_*xx*_(*f*) and *P*_*yy*_(*f*) indicate the power spectral densities of channels *x* and *y* for frequency *f*, respectively. The value of *P*_*xy*_(*f*) is calculated as follows:

$$P_{xy}(f)\;=\;\sum_{m\;=\;-\infty }^{+\infty }R_{xy}(m)e^{-i2\pi ft}$$ (2)

in which $R_{xy}(m)\;=\;E\{x_{n+m}y_{n}^{*}\},\;-\infty <n<\infty$ is the cross correlation of the *x* and *y* channels, and E{.} denotes the expectation of its argument. Values of *P*_*xx*_(*f*) and *P*_*yy*_(*f*) are calculated using similar equations. The values of *P*_*xy*_(*f*), *P*_*xx*_(*f*) and *P*_*yy*_(*f*) are calculated based on FFT analysis according to the periodogram method [49]. In practical terms, each pair of corresponding epochs from the *x* and *y* channels produces a value of cross power spectral density at each frequency bin. To calculate this value for each epoch, the FFT of the cross correlation function is calculated using one-second segments with 50% overlap using a Hamming window. The resulting set of FFTs are averaged according to the periodogram method [49]. Using Eq (1), the value of ImCoh is calculated for each frequency bin. This value is then averaged across all epochs for each participant. Then, to calculate the coherence deficit in the gamma band, the resulting values are averaged in the range of frequencies of the gamma band to obtain a single number. We refer to this number as the ImCoh value for the gamma band, and it serves as a measure of asynchrony between the *x* and *y* EEG channels in this band. In order to measure similar coherence deficit values for other oscillation bands such as delta (0.5–3.99 Hz), theta (4.0–7.99 Hz), alpha (8.0–12.99 Hz) and beta (13.0–29.99 Hz), the same calculation method is conducted using respective bandpass filters for each target band and averaging the ImCoh values over the frequencies in that band.

#### Statistical analysis of coherence deficit

A total of 30 coherence values (5 oscillatory bands times six pairs of electrodes: Fp1-Fz, Fp1-Cz, Fp1-Pz, Fz-Cz, Fz-Pz, Cz-Pz) were calculated. The Welch t-test [39] was applied, and p-values for all the ImCoh values were calculated. Due to the redundancy in these values (data from the six connections have some degree of correlation), we used the Benjamini-Hochberg correction method to control the FDR using an effective sample size calculation method described in Chapter 8 of [39]. ImCoh values with p-value < 0.05 were considered significant.

To assess the performance of the resulting significant ImCoh values in classifying mild AD patients and healthy participants, an SVM classifier was trained using the significant ImCoh values (beta and gamma ImCoh values for the Fz-Cz connection) as its features.

### Multimodal analysis

An objective of this study is to propose a multi-modal method for distinguishing mild AD patients from healthy participants based on the two olfactory-based data types. This is to suggest the most efficient combination of olfactory-based tests for diagnosing AD. To achieve this goal, an SVM classifier with a linear kernel was used to separate mild AD patients from healthy participants based on the significant components of the modified UPSIT test and the significant ImCoh values between the EEG electrodes, which were calculated for the beta and gamma frequency bands in the Fz-Cz connection. Since cognitive and olfactory performance declines with normal aging, two approaches were employed to address the presence of any confounding effect caused by the age of the participants. In the first approach, we trained our classifiers disregarding age as a feature. In the second approach, we measured the rate of change of each of our target features (UPSIT scores, beta and gamma ImCoh values) with age in the healthy group, and compensated the same feature in the AD group for the normal aging effect. Evaluation of classifiers based on single- or multimodal features was conducted using 5-fold cross-validation.

## Results

A p-value less than 0.05 was used as the cut-off value for the significance of all the stated results. The Scipy and Statsmodels packages were used for all statistical analyses described in the following.

### MMSE

Details of the MMSE results are shown in Table 2. This table includes the participant’s age, diagnosis state, total MMSE score as well as the MMSE score in each category. The p-value for each test category is also shown.

**Table 2:** MMSE results.

Diagnosis	Age	Edu	MMSE	O Time	O Place	Reg	AttCalc	DelRec	Name	Rep	Read	Write	VisSpat	Comm
Mild AD	75–80	6	19	4	4	3	0	0	2	1	1	1	1	2
Mild AD	75–80	0	16	2	5	3	0	2	2	1	0	0	0	1
Mild AD	80–85	3	18	4	5	3	1	0	2	0	0	0	0	3
Mild AD	85–90	0	12	2	3	3	0	0	2	1	0	0	0	2
Mild AD	70–75	4	15	2	5	3	0	0	2	0	0	0	0	3
Mild AD	85–90	0	16	3	4	3	0	0	2	1	0	0	0	3
Mild AD	65–70	5	12	3	2	3	0	0	2	0	0	0	0	2
Mild AD	65–70	0	18	2	3	3	0	3	2	1	0	0	0	3
Mild AD	70–75	6	19	5	3	3	3	0	2	0	1	1	0	1
Mild AD	80–85	8	17	2	5	3	0	0	2	0	1	1	0	3
Mild AD	60–65	5	11	3	2	3	0	0	2	0	0	0	0	3
Normal	75–80	6	27	5	5	3	4	2	2	1	1	0	1	3
Normal	70–75	5	30	5	5	3	5	3	2	1	1	1	1	3
Normal	65–70	6	26	5	5	3	4	2	2	0	1	0	1	3
Normal	65–70	12	30	5	5	3	5	3	2	1	1	1	1	3
Normal	70–75	0	21	5	5	3	1	2	2	1	0	0	0	3
Normal	60–65	14	29	5	5	3	5	2	2	1	1	1	1	3
Normal	65–70	0	21	5	5	3	1	1	2	1	0	0	0	3
Normal	55–60	4	27	5	5	3	2	3	2	1	1	1	1	3
Normal	75–80	0	23	4	5	3	4	2	2	1	0	0	0	3
Normal	70–75	0	26	5	5	3	5	1	2	1	1	0	0	3
Normal	70–75	6	26	5	4	3	2	3	2	1	1	1	1	3
Normal	65–70	0	21	5	4	3	1	3	2	0	0	0	0	3
Normal	60–65	12	28	5	5	3	3	3	2	1	1	1	1	3

Abbreviations used: Edu = education (years), O Time = orientation to time, O place = orientation to place, Reg = registration, AttCalc = attention and calculation, DelRec = delayed recall, Name = naming, Rep = repetition, Read = reading, Write = writing, VisSpat = visuo-spatial, Comm = commands. Data of excluded participants are not shown in the table.

### UPSIT

**Fig 2:**
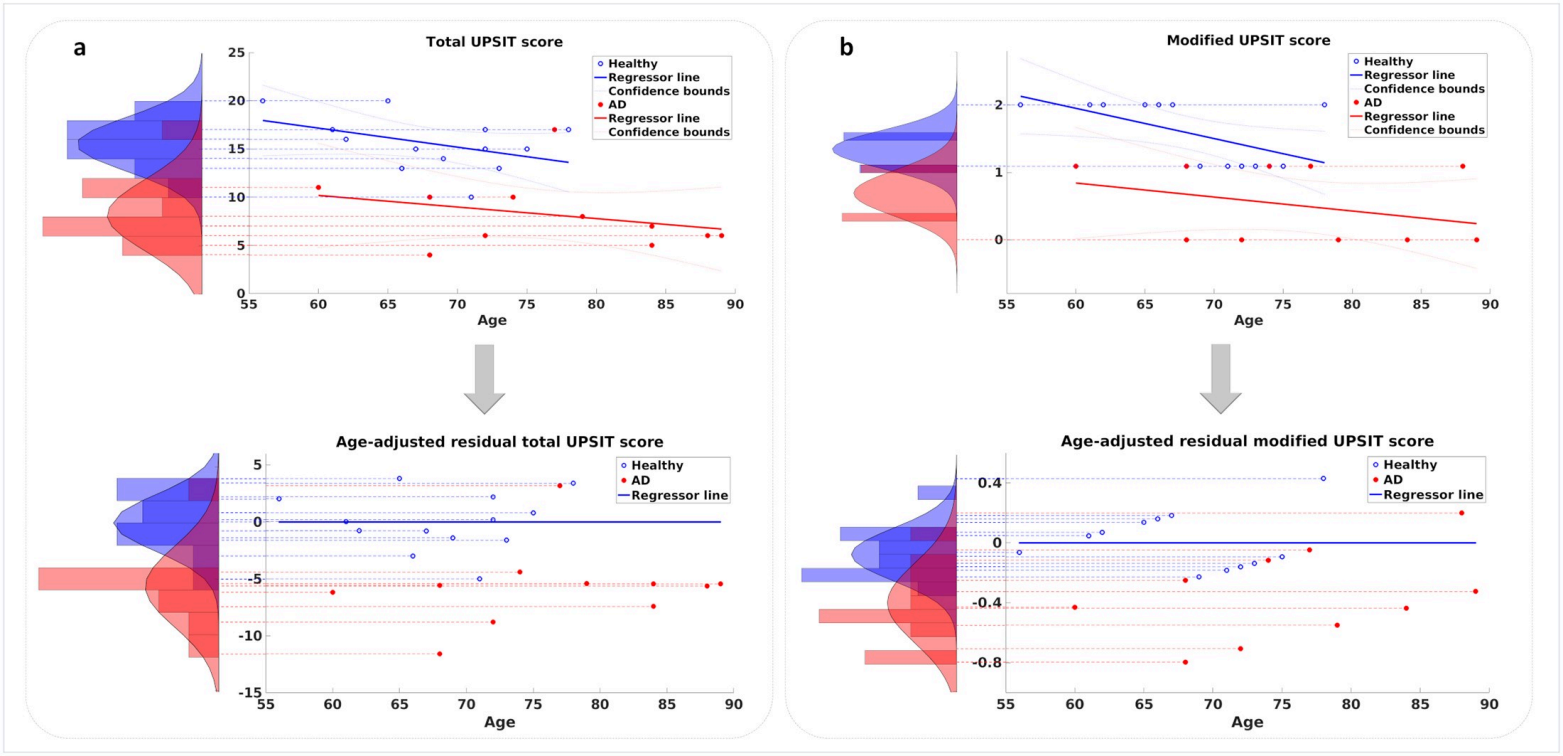
Total and modified UPSIT scores plotted versus age: a) Top: Regression of total UPSIT score with age. Distributions of total UPSIT scores for the two groups are shown as histograms. Bottom: Effect of age on total UPSIT score is removed and residual values of the two groups are histogrammed. b) Top: Regression of modified UPSIT score with age. Distributions of modified UPSIT scores for the two groups are shown as histograms. Bottom: Effect of age on modified UPSIT score is removed and residual values of the two groups are histogrammed. The 95% confidence intervals are also plotted in the top figures.

The UPSIT scores of the participants were used to classify them into the AD and healthy groups. Similar to other cognitive measures, the UPSIT score also displays a rate of decline with normal aging in healthy participants. As illustrated in Fig 2a, the two groups of participants demonstrate different rates of decline with aging. These rates can be inferred from the slopes of the regressor lines fitted to the scores of each group in this figure. While a noticeable drop is observed in the performance of the AD group relative to the healthy group, the rate of decline versus age remains similar. In order to assess the capability of the UPSIT score in discriminating between the AD and healthy groups, two approaches were employed for removing the effect of normal aging. First, age was disregarded and the UPSIT scores of each group’s members were used to create histograms that are shown in Fig 2a (top). Applying a single-feature SVM classifier to these histograms yields an accuracy of 87.5% in classifying the two groups. Second, age-adjusted scores were calculated by measuring residual distances of the scores from the regressor line fitted to the healthy group’s data. Fig 2a (bottom) displays age-adjusted UPSIT data for the two groups, from which the two illustrated histograms were produced, each capturing the residuals of a participant group from the healthy group’s age-regressed line (here shown as a horizontal line due to age adjustment). A single-feature SVM classifier was applied to these histograms and again, an accuracy of 87.5% was achieved in classifying the two groups. These results suggest a relationship between the olfactory functionality (represented by the UPSIT score) and the diagnosis of AD even when the effect of age confound is removed from the data.

As mentioned in the previous section, p-value was calculated for each odor to identify the most sensitive odorants. Two significant odors (p-value < 0.05) were identified (Grape and Chocolate). These odors, as well as their p-values, are shown in Table 3.

**Table 3:** P-values derived from t-test for significant UPSIT odors separating the two groups of participants.

Odorant	p-value
**Q6 (Grape)**	<0.05
**Q21 (Chocolate)**	<0.05

We then calculated the modified UPSIT score for each participant by summing the responses to the two significant odors. The resulting modified scores versus age are plotted in Fig 2b (top) for the two groups. Following a similar approach for removing the effect of age, the modified scores were employed to create the two histograms shown in Fig 2b (top), and employing a single-feature SVM classifier resulted in an accuracy of 70.8% in classifying the two groups based on the modified scores. This result displays a drop in the performance compared to the case when the entire UPSIT scores were used to classify the two groups. In the second approach, the effect of age was adjusted for by measuring the residual distances of all scores in the two groups from the regressor line fitted to the data of the healthy group. A single-feature SVM classifier was then applied to the resulting age-adjusted histograms shown in Fig 2b (bottom), yielding a classification accuracy of 87.5%. This result shows considerable improvement comparing with the accuracy of the age-disregarded approach, and is at par with the results obtained when the entire UPSIT scores were used to classify the two groups.

**Fig 3:**
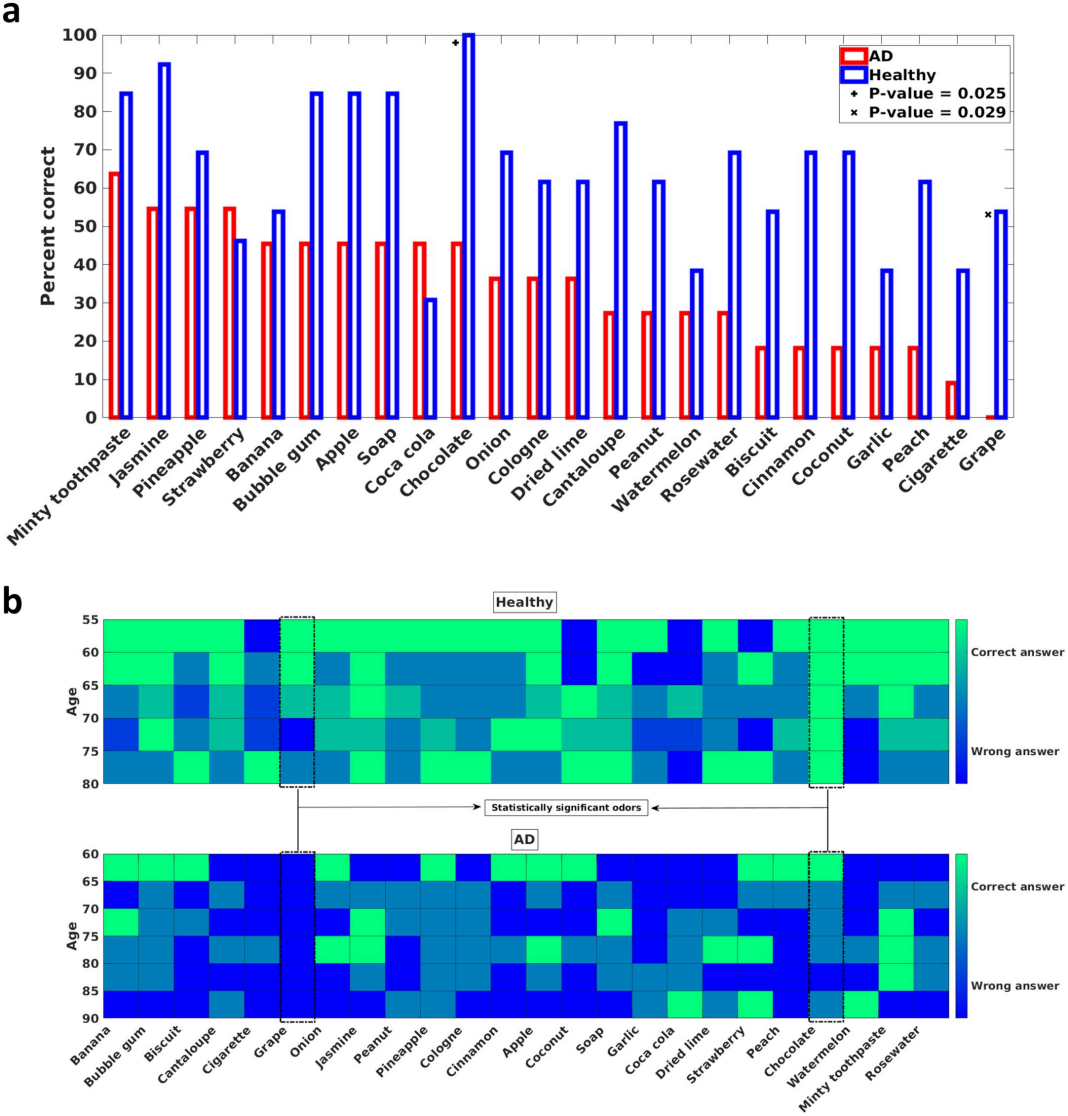
a) Percentage of correct answers for each UPSIT (Iran-SIT) odor identification question. The x-axis denotes the tested odors and is sorted from the left by the number of correct answers that the mild AD patients gave. Hence, the leftmost odor is the one that the mild AD patients identified most. The y-axis is the percentage of correct answers to each odor identification question in each group. Two odors that are statistically significant in distinguishing between mild AD patients and healthy participants are denoted by + and x marks. b) The number of correct answers for each UPSIT (Iran-SIT) odor identification question divided into five-year age bins. The shade of each bin denotes the number of correct answers. Green (light) pixels indicate that most participants in the corresponding age bin answered the question correctly, and the blue (dark) pixels suggest that most of the participants were unable to identify the presented odor. The upper diagram is for the healthy participants, and the lower diagram is for the mild AD patients. The two statistically significant odors are denoted by dashed boxes.

To analyze which odors can discriminate between mild AD patients and healthy participants and which odors can still be perceived by mild AD patients, two visualizations of the UPSIT results are presented in Fig 3. The two significant odors (Grape and Chocolate) are indicated in both plots. In Fig 3a, the percentage of mild AD patients or healthy participants who answered each UPSIT odor identification question correctly is plotted. It can be seen that more than half of mild AD patients correctly identified the smells of Minty Toothpaste, Jasmine, Pineapple, and Strawberry. Fig 3b shows the UPSIT results when the participants in each group are divided into five-year age bins.

### EEG coherence

**Fig 4:**
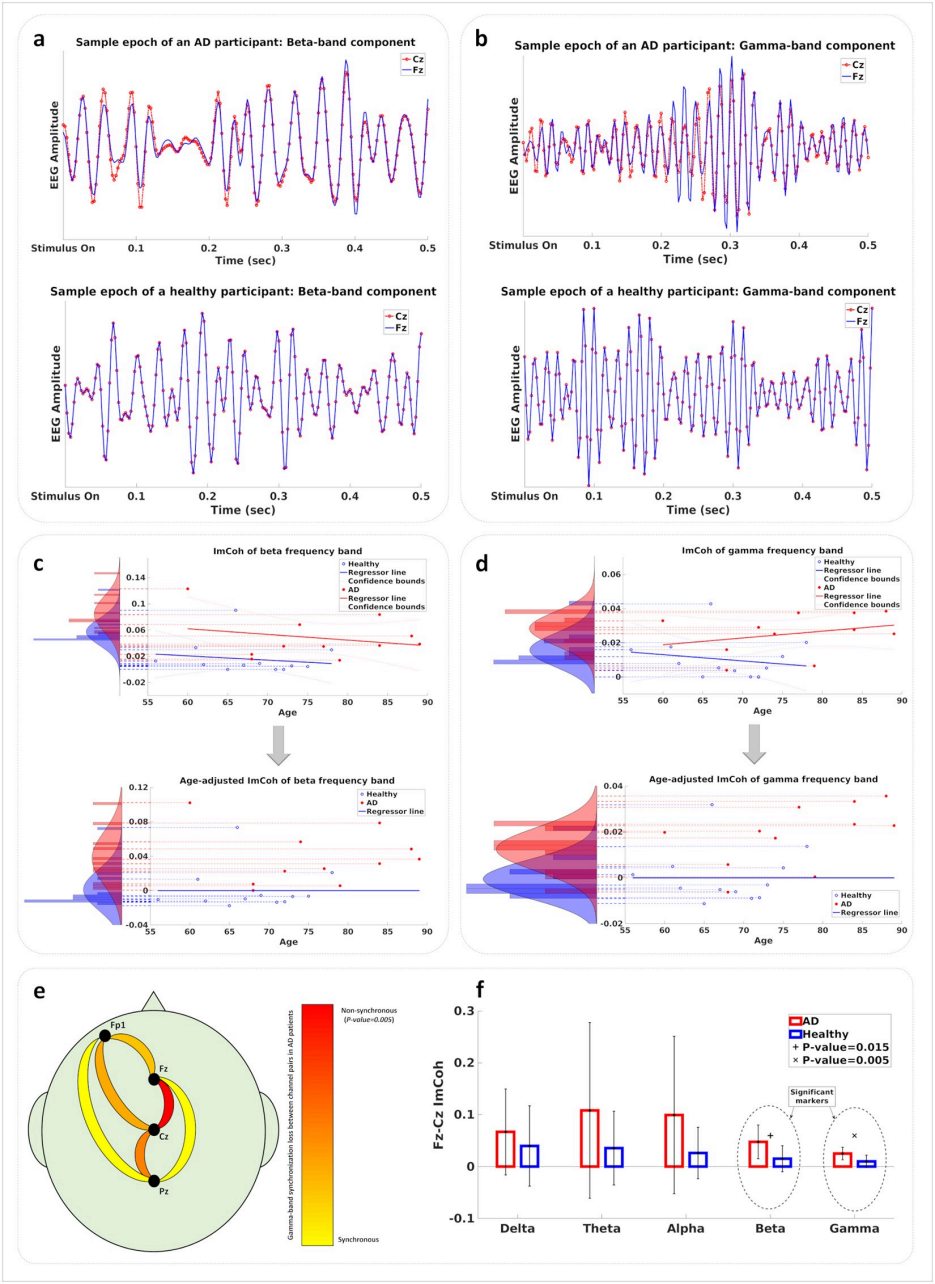
a) Beta-band component of a sample epoch from the data of the Fz and Cz electrodes for one AD (top) and one healthy (bottom) participant. b) Gamma-band component of a sample epoch from the data of the Fz and Cz electrodes for one AD (top) and one healthy (bottom) participant. c) Top: Regression of beta-band ImCoh value with age. Values for the two groups are shown as histograms. Bottom: Age-adjusted beta-band ImCoh values are shown as histograms for the two groups. d) Top: Regression of gamma-band ImCoh value with age. Values for the two groups are shown as histograms. Bottom: Age-adjusted gamma-band ImCoh values are shown as histograms for the two groups. e) Statistical significance of the gamma-band ImCoh value difference between mild AD patients and healthy participants. The Fz-Cz connection is the most significant connection with p-value < 0.05. f) The ImCoh value of the Fz-Cz connection measured in each frequency band. Statistically significant frequency bands are denoted by + and x marks.

The imaginary part of the coherence between each pair of EEG electrodes was calculated for each of the delta, theta, alpha, beta, and gamma frequency bands. This value is an indication of the lack of temporal synchrony between two signals. Fig 4a and 4b illustrate sample epochs in which 13–30 Hz and 30–40.5 Hz filters were respectively used to extract beta and gamma band components from the data of the Fz and Cz electrodes of one AD patient and one healthy participant. As these plots demonstrate, the beta and gamma components recorded by the two electrodes closely match each other in phase for the data of the healthy participant while the data of the AD patient contains intervals of clear out-of-phase behavior for these components. The overall measure used as the imaginary part of coherence (ImCoh) for a participant in a frequency band is calculated as the mean of the imaginary parts of coherence across the frequency band and averaged for all the frequent odor epochs used in the analysis.

The ImCoh values were calculated for each of the five frequency bands between each pair of electrodes in each participant’s EEG data. In order to assess the effect of age on the synchronization behavior of the brain as measured by pairwise ImCoh values, we employed a regressor fitting approach similar to the UPSIT analysis. Fig 4c and 4d show the regressor lines fitted to the beta and gamma ImCoh values for the healthy and AD groups. For each frequency band, histograms of ImCoh values were plotted for both cases when age is disregarded or when its effect is removed from the data according to the normal aging change. Each of these methods aims to remove any confound effect caused by normal aging from the data of AD patients. These age-removed beta- and gamma-band ImCoh values are used to train multi-modal classifiers (with and without the UPSIT scores), the results of which are described in the next section.

Statistical analysis indicated the ImCoh value between the Fz and Cz channels to possess the highest significance in separating the two groups of mild AD patients and healthy participants, with the gamma and beta ImCoh values having p-values less than 0.05. Fig 4e illustrates the relative significance of the six electrode-pair connections for the gamma-band ImCoh values, with the best p-value in the Fz-Cz connection. Fig 4f shows the mean and the standard deviation of the ImCoh values for all five frequency bands for the Fz-Cz connection across the AD and healthy groups. The beta and gamma ImCoh values for this connection offer significant discrimination power between the two groups.

### Multimodal analysis

We employed three multimodal classification methods based on the significant ImCoh values measured between the Fz and Cz channels in the beta and gamma frequency bands, and the total or modified UPSIT scores. Using single-feature classifiers based on the total or modified UPSIT scores resulted in classification accuracies reported in the UPSIT section earlier, which are included in Table 4 for comparison with multi-modal results. Table 4a includes classification results for the cases in which age was disregarded as a feature. In Table 4b, classification results are shown for the cases in which all measured features are age-adjusted according to the methods described earlier.

**Table 4:** Classification accuracy for different modalities and the multi-modal analysis based on significant components of each modality. a) Feature data were used for classification disregarding the age of the participants. b) Residuals of each feature relative to its corresponding healthy group regressor were used for classification.

**a) Classification Results (age-disregarded)**	**Accuracy (%)**	**AUC**
Total UPSIT	87.5%	0.91
Modified UPSIT	70.8%	0.84
Beta and Gamma ImCoh in Fz-Cz	75.0%	0.85
Beta and Gamma ImCoh in Fz-Cz + Total UPSIT	87.5%	0.9
**Beta and Gamma ImCoh in Fz-Cz + Modified UPSIT**	**91.7%**	**0.96**
**b) Classification Results (age-adjusted)**	**Accuracy (%)**	**AUC**
Total UPSIT	87.5%	0.90
Modified UPSIT	87.5%	0.85
Beta and Gamma ImCoh in Fz-Cz	79.2%	0.85
Beta and Gamma ImCoh in Fz-Cz + Total UPSIT	83.3%	0.87
**Beta and Gamma ImCoh in Fz-Cz + Modified UPSIT**	**91.7%**	**0.97**

The multimodal classifier which employs the beta- and gamma-band ImCoh values across the Fz-Cz electrode pair and the modified UPSIT score outperforms other classifiers in terms of accuracy. This means that the data collection protocol corresponding to this classifier only requires that the participants answer two questions in the UPSIT test and that the EEG data be recorded from 3 electrodes (Fz, Cz, Fp1) and a reference (A1), offering a convenient procedure for examining elderly participants. The performance of the proposed multimodal classifier is comparable to the MMSE-based assessment used routinely in clinical examinations.

In addition, the modified UPSIT classifier performs equally well compared to the total UPSIT score with age adjustment. This shows that even by using a limited number of selective odors, clinicians can obtain valuable indications about the state of a patient. However, a more extensive study is needed to further assess the validity of this claim.

**Fig 5:**
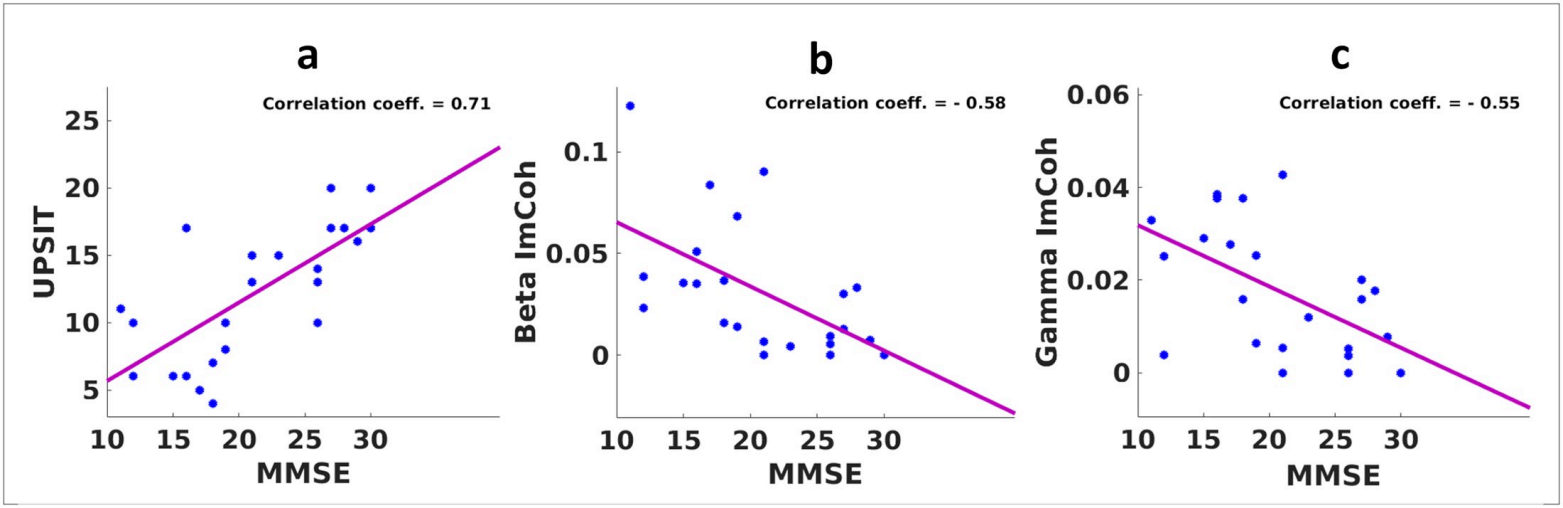
a) Correlation of the total UPSIT score and the MMSE score. b) Correlation of the beta-band ImCoh value and the MMSE score. c) Correlation of the gamma-band ImCoh value and the MMSE score.

In order to analyze how correlated the UPSIT score and the Fz-Cz beta and gamma ImCoh values are with the clinical MMSE score, these values for all participants are plotted versus MMSE scores in Fig 5. The Pearson’s correlation coefficient between the UPSIT and MMSE scores is 0.71 with a p-value of 0.0001, between the beta-band ImCoh value and the MMSE score is -0.58, and between the gamma-band ImCoh value and the MMSE score is -0.55.

## Discussion

Diagnostic methods such as PET, FDG-PET [50, 51] and MRI imaging and CSF sampling are expensive [52]. In particular, CSF sampling involves an invasive procedure, making it impractical for longitudinal studies requiring repeated sampling. The proposed olfactory-based methodology in this paper is convenient to conduct and has considerably lower cost. It is also more accommodating for elderly patients as both the behavioral and EEG tests only require a small number of measurements. It hence provides a viable solution for monitoring the progress of the disease in a patient over time, and offers opportunities for longitudinal research studies.

In this study, we showed that the grape and chocolate odors could discriminate between mild AD patients and healthy participants with good accuracy. Earlier studies have assessed the ability of different scents in similar tasks. For example, the study by Kjelvik et al. [2] identified some significant odors, which interestingly, chocolate was among them. A proposition for conducting olfactory-based studies would hence be to perform experiments in culturally-diverse groups of populations and identify the marker odors which are significant universally and those best suited for each culture or geography.

We also identified odors in our study which more than half of our mild AD patients could still perceive and successfully recognize. Understandably, a survey of a larger population is needed to identify such odors with more confidence. An interesting application for this set of scents would be to include them in olfactory assessment tests to discriminate between mild AD patients and individuals with anosmia or non-AD neurodegenerative diseases which can lead to the loss of olfactory functionality.

It should be noted that some odors could not be correctly identified by either of the healthy and AD groups. A reason for this lack of performance by the healthy participants might be the unfamiliarity of the study group with certain scents. For instance, both the AD and healthy groups were unable to identify the Coca-Cola odor because most Iranian elderly may have not experienced this smell in their daily life. These non-discriminating odors can be replaced in future sniffing kits by other scents to improve the performance of the test.

A known issue with interactive tests such as UPSIT is that the deficit in the perception of odors can be confounded by memory and cognitive impairment affecting the recall of the odor or its name. Nonetheless, the UPSIT experiment proved to possess sufficient discriminating power for the two subject groups in our study. Moreover, the fact that responses by the AD group differ considerably across the set of odors provides additional clues about the progression of the impairments associated with the olfactory perception through the advancement of the disease. The above points justify the inclusion of the UPSIT test in the studies on AD progression.

On the other hand, the mentioned confound issue is totally mitigated in the EEG experiment part of our study which does not involve verbal interactions with the subjects for identifying the names of the odors. Identifying biomarkers in the EEG signal in response to olfactory stimulation is indeed a major thrust of our study. The combined use of features from the UPSIT and EEG tests improved the classification accuracy by about 4 percent for the total UPSIT score and about 12 percent for the modified UPSIT score over the results of EEG alone. Another gain offered by the UPSIT experiment was to allow the examination of a set of 24 odors while the EEG experiment was conducted using 2 odors. The two significant odors identified in our analysis of the UPSIT data can be used to define a simple interactive olfactory screening test or as the two odors used in EEG recordings in the future.

The result of EEG-based olfactory assessment suggested that the coherence in the gamma and beta bands significantly differs between mild AD patients and healthy participants. This result is in agreement with the evidence about the roles that the gamma and beta bands play in cognitive functions of the brain, and their deficit resulting from the neurodegenerative effects of AD. The high-frequency gamma oscillations (30–100 Hz) and the beta oscillations (13–30 Hz) appear to be particularly well suited for the maintenance of functions in the brain that involve *binding* the processed data from different sensory modules or elements stored in the memory [53]. Multi-sensory data integration [54, 55], attentional sensory selection [56–58], working memory association [59], and generation of long-term memory through associations embedded as synaptic weight adaptation [60, 61], are all performed under the gamma and beta oscillatory regimes in the neuronal populations involved [53].

Employing high-frequency oscillations in functions that access and combine data from multiple sites in the brain is not a coincidence; the resolution that high-frequency oscillations such as the beta and gamma bands offer in their phase allows for fine-tuned coding of relative arrival times and latencies involved in accessing multiple threads of data and hence allows for effective input selectivity through high-precision control of spike timing [53, 62, 63].

Earlier studies of the neurodegeneration mechanisms affecting the olfactory perception in Alzheimer’s disease have indicated three possible candidates: 1) Disruption in the olfactory bulb functionality [19], 2) Weakening of the feedforward data circuitry in the medial temporal lobe [18, 19], and 3) Deficiency in the inhibitory feedback function of interneurons emanating from the frontal lobe [64, 65]. The EEG analysis results of our study establish an evidence for the significance of the third explanation, indicating loss of synchronous oscillations in the frontal lobe as an early marker of AD.

**Fig 6:**
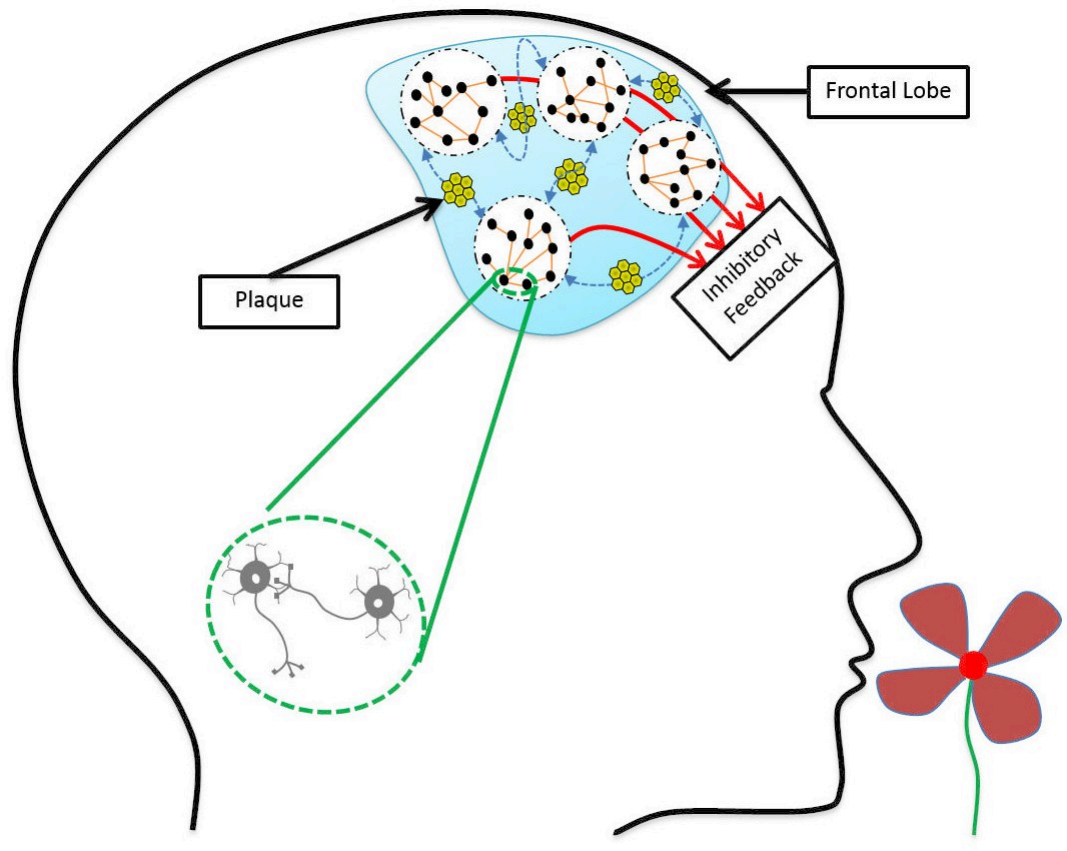
Deposition of amyloid-β plaques in the frontal lobe leads to the weakening of neuronal connections. Neuronal populations are still working, but they are not synchronous enough to send effective inhibitory feedback to earlier processing layers.

Desynchronized neural activity and disruption of gamma oscillations have been observed in both human mild AD patients [64, 66–68] and the AD mouse models [69–71]. As neuronal connectivity is affected by the accumulation of amyloid-β in the extracellular space [72], considering such aggregation across a large population of neurons allows to model AD as a network operation deficiency problem [73]. In such models, large inhibitory circuits operating under high-frequency regimes turn into subpopulations that may produce these oscillations without synchrony with each other. As illustrated in Fig 6, amyloid-β plaques are accumulated between neuronal populations in the frontal lobe, resulting in desynchronized inhibitory feedback to earlier layers. In [74] the deficit in coherence between oscillations measured by EEG electrodes across the scalp in different frequency bands has been proposed as a diagnostic marker of dementia caused by Alzheimer’s disease.

While it is still a matter of debate whether these deficits in high-frequency oscillations are a consequence of the underlying disease progression or that they indeed play a causal role in inducing more biological changes that promote the disease [72], the deficit in the high-frequency oscillations can be associated on the functional level with the lowered *binding* activity in the cortex, causing the known symptoms of AD such as cognitive decline and dementia. Our study revealed the significance of the gamma and beta band coherences in separating mild AD patients and healthy participants and showed the difference to be more significant across the spatial range measured by the Fz and Cz electrodes. This is the scalp region close to the cortical areas known to be involved in many cognitive functions.

Our study showed that the olfactory deficit could be a fairly accurate marker for AD when behavioral assessment results are combined with the coherency results of the EEG recording. The accuracy of the proposed multi-modal classifier is significantly above the chance level (91.7%). Even if we develop a classifier based on the MMSE scores—which is based on tests that directly evaluate the participant’s memory and cognition—we may not always reach 100% accuracy. A common issue in performing the MMSE test is that some of its questions require reading and writing skills and therefore, illiterate subjects cannot get any scores from those parts. Also, running the test requires interaction between the participant and the memory specialist, increasing the probability of introducing bias during the test. Unlike MMSE, the proposed method in this study requires much less interaction and the behavioral olfactory assessment (UPSIT) test can be carried out even by the participants themselves if they have the ability to read the questions.

### Limitations

We have demonstrated the suitability of our methodology in identifying mild AD patients in a limited study on the Iranian population. The proposed method needs to be applied to a bigger population using a larger set of odorants in order to further validate and map out the usefulness of olfactory-based biomarkers in the diagnosis of AD. Furthermore, a larger-cohort study can reveal the effectiveness of our methodology when patients suffering from non-AD dementia or moderate and severe AD patients are also present in the study.

As odor perception is a culture-dependent phenomenon, the exact results derived in our study may not be directly applicable to different populations. A more comprehensive study comprising of populations of participants from different cultural backgrounds is needed to verify the validity of the proposed approach in general, and the significance of the individual scents or other features used in our regressors and classifiers in particular.

A related notable remark is that while the results of the smell identification test (UPSIT) may have strong cultural dependencies, the EEG-based coherence analysis may prove to be a relatively more robust procedure. This is due to the fact that higher-level functions of the brain are represented in the coherence values measured in the EEG analysis, and some level of abstraction from the particular smells that are perceived may be represented in these measurements.

Another limitation of the smell identification test is that successfully answering questions in it involves both the perception of the presented odor as well as its recognition through a memory recall process. This indicates an inherent ambiguity in this test between a lack of perception of the presented smell and failure to identify the name of the odor which may have indeed been perceived. The EEG-based olfactory assessment is advantageous in this aspect to the UPSIT test as it mainly focuses on the perception ability and not the identification or naming functions.

### Extensions

One interesting extension of this work is to repeat the EEG-based experiments using the two odors (chocolate and grape) which were identified in the olfactory recognition task as significant and compare the results of the coherence analysis with the current results. As these two odors best separate the two groups of participants, it is interesting to see any gains their usage may provide to the EEG-based coherence results.

An essential extension to the EEG analysis is to examine other approaches, such as the difference between the responses to the two odorants within each group of mild AD patients and healthy participants. There are two possible ways to derive these differences. One is to repeat the current coherence-based approach separately for each of the odorants. The other is to make the comparison directly in the temporal domain of the recorded EEG data after artifact and noise removal. Possible advantages of these odorant-differential analyses may include the additional dimension that they provide as the difference in responses to the two presented odors. To perform this extension, it is better to conduct the experiments with a sequence of odors in which both odorants are presented randomly with equal probabilities so the number of epochs related to the two odorants would be comparable.

Another possible domain for extending the EEG-based olfactory test is to repeat the experiment for each participant in more than one session and use different pairs of odorants in each experiment. This allows for studying the sensitivity map of each participant relative to different odors. Running an experiment with more than two odorants presented by the same olfactometer is a possibility but requires modifications in the design of the olfactometer.

On the cohort design side, a necessary extension is to recall the participants for another round of experiments after a period of six to twelve months and perform longitudinal analyses to study the correlation between the olfactory decline and the progress of the disease in each patient. Another critical extension that can further evaluate the specificity power of the proposed approach is to include a third participant group consisting of non-AD MCI patients and examine the single- and multi-modal analysis methods of the current study in discriminating the three groups from one another. These two latter extensions are within the scope of our on-going data collection campaign. Any additional results achieved in each of these extended studies will be provided in future reports.

## Conclusions

In this paper, we have demonstrated the efficacy of an inexpensive methodology for evaluating the olfactory deficit in the elderly population for being utilized as a marker of AD with good accuracy. Our proposed approach combines behavioral olfactory data with EEG measurements to yield an accurate assessment of the participant’s state.

Statistical analysis of the results of the smell identification test yields two odors (Chocolate and Grape) as significant (p-values < 0.05) from a set of 24 odorants. The EEG coherence analysis indicates the gamma and beta bands to be significant (p-values < 0.05) in the link between the Cz and Fz channels, with the gamma band possessing a higher significance. The proposed multi-modal classifier yields an accuracy of 91.7% in separating mild AD patients from healthy participants.

The accessibility and low cost of the proposed procedure allow for large-scale screening of AD in different geographical regions, a looming necessity across the world as the aging population is rapidly expanding. Furthermore, athe results of this work can provide researchers with new insights about the relationship between AD progression and olfactory deficit and can lead to new treatment methods based on olfactory stimulation.

## Supporting information

S1 Appendix (DOCX)
