# Supplementary material for: Olfactory response as a marker for Alzheimer’s disease: Evidence from perceptual and frontal lobe oscillation coherence deficit
Source: PLoS One. 2020 Dec 15;15(12):e0243535. doi: 10.1371/journal.pone.0243535 (PMC7737889; doi:10.1371/journal.pone.0243535)
Supplement: S1 Appendix — (DOCX) [file pone.0243535.s001.docx]

**S1 Appendix**

**S1 Table. UPSIT (Iran-SIT) Test Questions**

The list of Iran-SIT odor smelling kit questions is shown in the table below (correct answers, i.e. the presented odors, are denoted in boldface).

|  | **A** | **B** | **C** | **D** |
| --- | --- | --- | --- | --- |
| 1 | Gasoline | Cherry | **Banana** | Garlic |
| 2 | Pizza | Cucumber | Alcohol | **Bubble Gum** |
| 3 | Cologne | **Biscuit** | Tuberose | Orange |
| 4 | Cinnamon | Vinegar | **Cantaloupe** | Kebab |
| 5 | Peach | Saffron | **Cigarette** | Tangerine |
| 6 | **Grape** | Roasted Seed | Parsley | Fried Chicken |
| 7 | Melon | Rose | Coffee | **Onion** |
| 8 | Lemon | **Jasmine** | Smoke | Fish |
| 9 | **Peanut** | Celery | Apple | Cardamom |
| 10 | Tea | Tobacco | **Pineapple** | Gas |
| 11 | Cake | Sour Orange | Black Pepper | **Cologne** |
| 12 | **Cinnamon** | Pomegranate | Egg | Washing Machine Powder |
| 13 | Olive | Sewage | **Apple** | Butter |
| 14 | **Coconut** | Rosewater | Sausage | Smoke |
| 15 | Persian Herb Stew | **Soap** | Lemon | Watermelon |
| 16 | Chocolate | Saffron | Cucumber | **Garlic** |
| 17 | Basil | Pear | Fish | **Coca Cola** |
| 18 | Hami Melon | **Dried Lime** | Honey | Grapes |
| 19 | Olive | **Strawberry** | Leather | Thyme |
| 20 | Peanut | **Peach** | Coffee | Pizza |
| 21 | **Chocolate** | Sour Orange | Vinegar | Sewage |
| 22 | Garlic | Cigarette | **Watermelon** | Bread |
| 23 | Rice | Pistachio | Pepper | **Minty Toothpaste** |
| 24 | **Rosewater** | Orange | Gasoline | Cheese |

x

**S1 Figure. Procedure for determining significant UPSIT odors in classifying AD vs healthy control participants.**


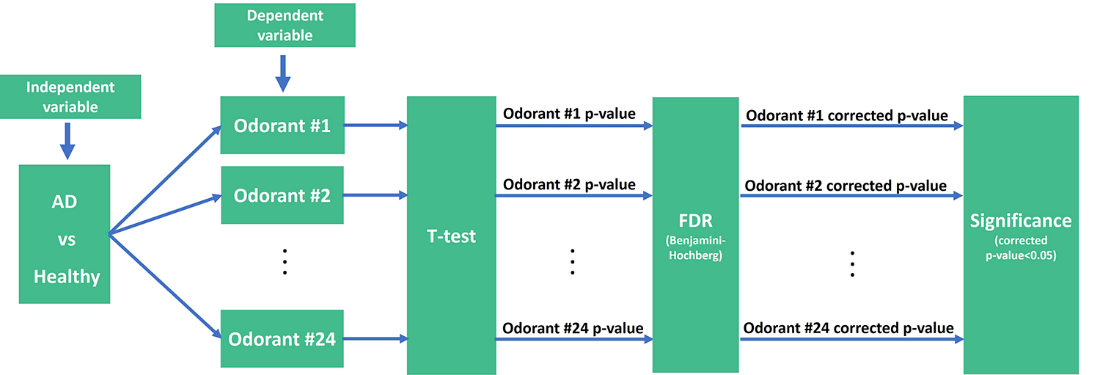


**S2 Table. UPSIT (Iran-SIT) FDR Corrected P-values**

The table below shows values behind the p-values derived through running t-test and the Benjamini-Hochberg method for controlling the FDR. The results indicate the difference in the perception of odorants by the two groups of AD and healthy control participants.

| Odorant number | 1  (Banana) | 2  (Bubble Gum) | 3  (Biscuit) | 4  (Cantaloupe) | 5  (Cigarette) | 6  (Grape) | 7  (Onion) | 8  (Jasmine) | 9  (Peanut) | 10  (Pineapple) | 11  (Cologne) | 12  (Cinnamon) |
| --- | --- | --- | --- | --- | --- | --- | --- | --- | --- | --- | --- | --- |
| Corrected p-value | 0.697 | 0.096 | 0.155 | 0.065 | 0.182 | **0.028** | 0.186 | 0.096 | 0.182 | 0.549 | 0.334 | 0.064 |
| Odorant number | **13**  (Apple) | **14**  (Coconut) | **15**  (Soap) | **16**  (Garlic) | **17**  (Coca Cola) | **18**  (Dried Lime) | **19**  (Strawberry) | **20**  (Peach) | **21**  **(Chocolate)** | **22**  (Watermelon) | **23**  (Minty Toothpaste) | **24**  (Rosewater) |
| Corrected p-value | 0.096 | 0.064 | 0.096 | 0.374 | 0.549 | 0.334 | 0.697 | 0.096 | **0.024** | 0.634 | 0.341 | 0.096 |
